# Supervised classification in the presence of misclassified training data: a Monte Carlo simulation study in the three group case

**DOI:** 10.3389/fpsyg.2014.00118

**Published:** 2014-02-28

**Authors:** Jocelyn Holden Bolin, W. Holmes Finch

**Affiliations:** Department of Educational Psychology, Teachers College, Ball State UniversityMuncie, IN, USA

**Keywords:** supervised classification, training data, misclassification, classification and regression trees, random forests, discriminant analysis

## Abstract

Statistical classification of phenomena into observed groups is very common in the social and behavioral sciences. Statistical classification methods, however, are affected by the characteristics of the data under study. Statistical classification can be further complicated by initial misclassification of the observed groups. The purpose of this study is to investigate the impact of initial training data misclassification on several statistical classification and data mining techniques. Misclassification conditions in the three group case will be simulated and results will be presented in terms of overall as well as subgroup classification accuracy. Results show decreased classification accuracy as sample size, group separation and group size ratio decrease and as misclassification percentage increases with random forests demonstrating the highest accuracy across conditions.

## Introduction

The need to classify individuals into one of two or more observed groups based upon a set of predictor variables is very common in the social and behavioral sciences (Zigler and Phillips, 1961; Arabie et al., 1996; Keogh, 2005). In many applications, an initial training sample of individuals from the population, for whom group membership is known, is used in conjunction with a statistical tool (e.g., discriminant analysis) in order to create a predictive model, which is then applied to individuals for whom group membership is not known. In this way, a predicted group membership can be obtained for the new individuals, and presumably then used to make decisions regarding the individual. For example in the field of education, statistical methods such as discriminant analysis are used to develop mechanisms for the identification of individuals on the basis of language impairment (Kapantzoglou et al., 2012), student instruction preferences (Clayton et al., 2010), disability status (Dunn, 2007; Lillvist, 2010; Mammarella et al., 2010) anxiety disorders (Clark et al., 1994), and career choice (Russell, 2008). While some of these categories are directly observable (for example, career choice and student instruction preference) such that initial placement of individuals in the training sample into categories can be made with some confidence, other category types are less concrete (i.e., anxiety disorder and disability status), so that the initial placement of those in the training sample may not be uniformly accurate. In turn, such errors would shed some doubt on the legitimacy of the classifications. Due to the widespread use of classification methods, as well as the potential for errors in the initial classification of members in the training sample, and the important decisions and consequences often associated with the group into which an individual might be placed using these methods (Sireci et al., 1999; DiStefano and Morgan, 2011), it becomes of utmost importance to determine not only which statistical classification methods are most accurate for the situation at hand but also which are most accurate when initial “true” group classifications may be questionable.

As has been demonstrated previously, statistical classification methods are greatly affected by the characteristics of the data under study. Previous research indicates classification accuracy generally increases with increased sample size (Holden and Kelley, 2010; Holden et al., 2011; Pai et al., 2012), discrepancy in group size (Lei and Koehly, 2003; deCraen et al., 2006; Holden and Kelley, 2010; Holden et al., 2011), group separation (Blashfield, 1976; Lei and Koehly, 2003; Holden and Kelley, 2010; Holden et al., 2011), and number of variables used in the classification (Breckenridge, 2000). Assumption violations (Lei and Koehly, 2003; Rausch and Kelley, 2009), outliers and presence of multicollinearity (Pai et al., 2012) generally lead to decreased classification accuracy. It should be noted, however, that the research on effects of data and distribution characteristics on classification accuracy has mainly focused on more traditional forms of statistical classification (Holden et al., 2011), namely discriminant function analysis, logistic regression and k-means cluster analysis. There is a small but growing body of literature supporting the use of newer and more sophisticated statistical classification and data mining techniques. Previous research has shown that these newer classification techniques are often more accurate than standard discriminant analysis (Reibnegger et al., 1991; Yoon et al., 1993; Curram and Mingers, 1994; West et al., 1997; Grassi et al., 2001; Holden et al., 2011). There is still little consensus when comparing the accuracy of these techniques against one another, however, and relatively few simulation studies comparing classification accuracy of these techniques exist.

In addition to the aforementioned characteristics of the data, statistical classification can also be complicated by initial observed group misclassification (Lachenbruch, 1966, 1974, 1979; McLachlan, 1972; Chhikara and McKeon, 1984; Grayson, 1987; Hofler, 2005; Holden and Kelley, 2010; Balamurali and Kalyanasundaram, 2011; Chen et al., 2011; Sal y Rosas and Hughes, 2011). Misclassification can be thought of as a type of measurement error (Betebenner et al., 2008; Ozasa, 2008) and can take several different forms. For example a distinction can be made between classification that occurs completely at random and misclassification that is non-random, occurring systematically based on the relative location of the point on its distribution (Lachenbruch, 1966, 1974, 1979; Chhikara and McKeon, 1984; Holden and Kelley, 2010). Misclassification can also be differential or non-differential. Non-differential misclassification occurs when the probability of misclassification is the same for all study groups. Differential misclassification occurs when the probability of misclassification differs between study groups (Ozasa, 2008). Misclassification can also happen at either the exposure (for example, was the individual in the treatment or the control group) or the outcome level (Hofler, 2005; Ozasa, 2008; Sal y Rosas and Hughes, 2011) [for example is the student academically proficient or not (Betebenner et al., 2008)]. The focus of this study will be on non-random or what we will term *systematic outcome misclassification* of training data, which is analogous to outcome misclassification resulting from artificial cut-point placement. From a statistical perspective, this occurs when the probability of a case being misclassified depends on that case's relative position in the distribution of the variable used to classify. For example, when an artificial cut-point is used with a measure in order to create groups using a continuous variable, individuals with scores closer to the cut-point are more likely to be misclassified whereas individuals with values further from the cut-point are less likely to be misclassified (Lathrop, 1986; Dwyer, 1996). Thus, if a specific score on a test is used to identify students at risk for academic problems, some individuals will very likely be misclassified (DiStefano and Morgan, 2011), with those having scores just above or just below the cut value most likely to be so. To illustrate, the No Child Left Behind act (2001) defined school quality in terms of the percent of examinees scoring at or above proficient. Generally, proficiency categorizations in educational assessment are determined by use of a cut point on a criterion referenced test (Sireci et al., 1999; DiStefano and Morgan, 2011). This process of categorization is very much susceptible to measurement error and measurement error of this type has been demonstrated to negatively impact classification accuracy and accuracy of performance level measures (Betebenner et al., 2008). If these already potentially flawed proficiency categorizations are then used in combination with a statistical classification procedure to identify students in future samples who may be at risk, such initial misclassification could be very problematic.

## Discussion of prediction methods

Indeed, several studies have shown that systematic misclassification in the two group case is detrimental to classification accuracy of traditional supervised classification methods (i.e., Discriminant analysis) (Lachenbruch, 1966, 1974, 1979; McLachlan, 1972; Chhikara and McKeon, 1984; Holden and Kelley, 2010). There is little research, however, investigating the impact of systematic training data misclassification when three true groups are present, or for newer classification and data mining techniques. Therefore, the purpose of this paper is to fill these gaps in the research and investigate the impact of systematic training data misclassification on three group misclassification using both traditional classification [Discriminant Function Analysis, both linear (LDA) and quadratic (QDA) and Logistic Regression (LR)] and newer statistical classification and data mining techniques. [Classification and Regression Trees, (CART), Generalized Additive Models (GAM), Neural Networks (NNET), Mixture Discriminant Analysis (MIXDA), and Random Forests (RF)]. Following is a very brief discussion of each of these methods. The interested reader is encouraged to obtain more in depth descriptions in the references provided below.

### Discriminant analysis

Linear Discriminant Analysis is a very widely used and effective technique for developing classification algorithms to differentiate two or more groups based on one or more predictors (Huberty and Olejnik, 2006). LDA finds weights for each predictor variable in a set such that the linear combination of these predictors maximally separates the groups from one another. These linear combinations can be used to determine category membership for each observation in the original training data or in a cross-validation sample, using the following equation:

(1) $$C_{ik}=c_{k0}+\sum_{j\;=\;1}^{J}c_{jk}x_{ijk}+\text{ln}\;(\frac{n_{k}}{N})$$

where

*C_ik_* = classification score for subject *i* in group *j*

*c*_*k*0_ = constant for group *k*

*c_jk_* = coefficient of variable *j* for group *k*

*x_ijk_* = value of variable *j* for subject *i* in group *k*

*n_k_* = sample size for group *k*

*N* = total sample size

Individuals are classified into the group for which they have the classification score,*C_j_*.

One drawback of linear discriminant analysis is that it constrains the group covariance matrices to be equal. In practice, constraining data to have this covariance structure may be too strict of an assumption. For such cases, a related form of discriminant analysis, quadratic discriminant analysis may be used (Huberty and Olejnik, 2006). Estimation in QDA is essentially the same as that for LDA except QDA allows each group to have their own separate covariance matrix, thus providing for a slightly more flexible structure.

### Logistic regression

In addition to discriminant analysis, another very popular method for group prediction is Logistic Regression (LR; Agresti, 2002), which models group membership as the log of the odds of being in one group versus another (the logit), as a function of the predictor variables.

(2) $$\text{ln}\;(\frac{\pi_{ik}}{1-\pi_{ik}})=\beta_{0}+\sum_{j\;=\;1}^{J}\beta_{j}x_{ij}$$

where

π_*ik*_ = probability of person *i* being in group *k*

β_0_ = intercept

β_1_ = coefficient for variable *j*

*x*_*ij*_ = value of variable *j* for person *i*

LR assumes a linear relationship between these predictors and the logit. While it is possible to incorporate non-linear terms into the model through the use of interactions among the independent variables, or by raising these variables to a power, such decisions must be made *a priori* by the researcher. For the purposes of prediction, the model in (2) is used to obtain the probability of an individual being in group *j*, with the predicted membership being *j* if the probability of membership is greater than 0.5. It should be noted that while 0.5 is the typical value here, other values for this threshold could be used if the research situation called for such. Otherwise the observation is predicted to belong to the other group.

### Generalized additive models

While LR and LDA are very popular and do have the advantages of being relatively simple, and having well understood and effective estimation algorithms, they also have some distinct disadvantages in practice. Perhaps foremost among these is that they are limited to addressing situations in which the relationship between the predictors and response (group membership) are linear in nature, unless the researcher explicitly includes non-linear terms in the form of interactions or squared main effects. Obviously, when the relationship between one or more predictors and the group is not linear in nature, this model will be of somewhat limited utility (Wood, 2006). An alternative methodology that is available to researchers working in such conditions are Generalized Additive Models which allow for the linking of the outcome (e.g., group membership) with one or more independent variables using smoothing functions such as splines or kernel smoothers (Hastie and Tibshirani, 1996). In the context of a binary outcome variable, GAM takes the form:

(3) $$\text{ln}\;(\frac{\pi_{ik}}{1-\pi_{ik}})=\beta_{0}+\sum_{j\;=\;1}^{J}f_{j}x_{ij}$$

where

π_*ik*_ = probability of person *i* being in group *k*

β_0_ = intercept

*f*_*j*_ = smoothing function for independent variable *j*

*x*_*ij*_ = value of variable *j* for person *i*

The selected scatterplot smoothing technique is employed with the goal of minimizing the penalized sum of squares (PSS) criterion to identify the optimal set of weights (*B*) for a given problem. The PSS is similar to the standard sum of squares that is minimized in regression, with the addition of a penalty term for model complexity, expressed as the number of model parameters. The desired degree of smoothing is controlled by the researcher through the use of tuning parameter, λ, which is greater than or equal to 0. A value of 0 results in an unpenalized function and relatively less smoothing, while values approaching 8 result in an extremely smoothed (i.e., linear) function relating the outcome and the predictors. Based upon empirical research, a recommended value for λ is 1.4 (Wood, 2006), and as such will be used in this study. The most common smoothing function used with GAM's (and the one used in this study) is the thin plate regression spline (Wood, 2006). This introduction to GAMs was intended to be brief. The interested reader is referred to any of several excellent sources that describe GAM in more detail, including Hastie and Tibshirani (1996), Wood (2006), and Hastie et al. (2009).

### Classification and regression trees

A potential drawback of GAM is that while it does not restrict the relationships between predictors and response to be linear, it does rely on an additive model, and the researcher must, to some degree, prespecify the nature of the non-linear relationships by indicating the type of spline to use and the degree of smoothing. One set of methods for regression and classification that does not require the researcher to prespecify anything about the nature of the model, except the predictor variables are those based on recursive partitioning. One of the earliest such method to be described in the literature is classification and regression trees, which were first outlined in detail by Breiman et al. (1984). CART is a non-parametric method (not assuming any particular form of the relationship linking predictors and the outcome variable) that arrives at predicted group membership given a set of predictors by iteratively dividing individual members of the sample into ever more homogeneous groups, or nodes, based on values of the predictor variables.

CART begins building a tree by placing all subjects into a single node. It then searches the set of predictor variables to find the value of one of those by which it can divide the observations and create two new nodes that are as homogeneous as possible with respect to the outcome (grouping) variable. Once this optimal split in the initial, or root node is found and the individuals are moved into one of the two resulting daughter nodes, the predictors are once again searched for the optimal split by which the observations can be further divided into ever more homogeneous groups, again with respect to the group variable. This process continues until further division does not yield decreases in within node heterogeneity, at which point the tree stops growing. At each split, CART seeks to minimize the deviance in the resulting nodes, which can be expressed as:

(4) $$D_{m}=-\text{2}\sum_{k\;=\;1}^{K}\sum_{m\;=\;1}^{M}n_{mk}\text{ln}\;(p_{mk})$$

where

*n*_*mk*_ is the number of subjects from group *k* in node *m*

*p*_*mk*_ is the proportion of subjects from group *k* in node *m*

The sum of the deviance across nodes is

(5) $$D=\sum_{m\;=\;1}^{M}D_{m}$$

This statistic serves as a measure of the homogeneity of the tree as a whole. At the conclusion of the tree growing process, the final or terminal nodes are then categorized as belonging to the group which has the largest value of *p*_*mk*_ therein.New cases can then be introduced to the tree in order to obtain a group classification. Their predicted group is equal to the plurality group for the terminal node into which they are placed based on the predictor splits identified by CART.

### Random forests

A major weakness of CART that has been identified in the literature is the potential instability of trees across samples from the same population, due to its sensitivity (Breiman, 2001). At the same time, research has also shown that an individual CART tree does produce unbiased predictions, so that averaged over a number of individual trees, the resulting predictions for an individual should be quite accurate (Bauer and Kohavi, 1999; Dietterich, 2000). Given this fact, researchers have developed alternative methods for developing predictive models based upon the recursive tree model outlined above. These two methods, Bagging (Breiman, 1996) and Random Forests (RF; Breiman, 2001) each rely on bootstrap resampling to overcome the aforementioned problems with CART. Specifically, both methods select a large (e.g., *B* = 1000) number of bootstrap samples of the sampled individuals, and apply CART to each of these. These bootstrap samples can either be drawn with replacement and be the same size as the original or without replacement and represent subsets of the original sample. The results of the *B* trees are then averaged to ascertain both variable importance information, and to predict an individual's group membership. The two techniques differ in that Bagging makes use of the entire set of predictors when constructing each tree, while RF applies bootstrapping (sampled without replacement) to the predictors as well as the sample, using a subset for building each tree. Thus, for each RF tree *B* bootstrap samples of subjects and predictor variables are used. Because the trees used by RF are even more diverse than those used in Bagging, it can be shown that its averaged results are also less sensitive to sample specific variation and thus potentially more generalizable (Breiman, 2001). In addition, although not a focus of the current study, by relying on bootstrapped samples of predictor variables RF provides more information than either Bagging or CART regarding the true importance of individual predictors in terms of correct group prediction. Given these advantages, RF will be the method of ensemble prediction of primary interest in this study.

With regard to prediction of a categorical outcome variable for a new sample using RF, the standard method of using a training sample to grow the trees, and a cross-validation sample to test it, much as was described above for CART, can be used. In this way, each tree is applied to each cross-validation case and the final predicted category in each such application is recorded. After all of the trees have been applied, each individual is placed into the category for which they have the most votes; i.e., into which they have been placed most frequently by the set of trees.

### Mixture discriminant analysis

MIXDA is an extension of LDA in which membership in each known group is modeled as a mixture of Gaussian distributions, rather than a single homogeneous distribution (e.g., Hastie and Tibshirani, 1996). The MIXDA model represents each observed group by the multivariate mean of predictors (centroid), as in LDA, but also allows latent classes to exist within each known group. In other words, existing groups (e.g., females and males) may consist of two or more unobserved groups of individuals. Thus, unlike LDA, MIXDA models predict group membership as a function of a mixture rather than a homogeneous distribution of the predictors. Parameter estimation in MIXDA relies upon the Expectation Maximization (EM) algorithm (Dempster et al., 1977), which yields subgroup means, common or group specific variance, the within group mixing proportions, and the between group mixing proportions, all of which are obtained from the training data. Predicted membership in the known classes can then be obtained for a cross-validation sample by simply applying the MIXDA model parameters to the predictor variable values for each of the new observations, much as with LDA.

At present, MIXDA is relatively unknown in the social sciences. However, MIXDA has been used successfully in a wide variety of research applications, such as biology, wildlife management (Britzke et al., 2011), and computer science (Kleinsmith et al., 2006). For example, Britzke et al. (2011) did a comparison of classification techniques for the acoustic identification of bats and found that MIXDA yielded the highest classification accuracy. Similarly, compared classification techniques for single-cell differentiation and found MIXDA to exhibit high prediction accuracy as well. MIXDA has also been shown to be particular useful when the predictor variables used are non-normal (Rausch and Kelley, 2009), and when attempting to classify relatively small groups when other groups in the sample are much larger (Rausch and Kelley, 2009; Holden et al., 2011).

### Neural networks

The final group prediction method examined in this study is Neural Networks (e.g., Garson, 1998; Marshall and English, 2000). NNETs identify predictive relationships between a categorical outcome and one or more predictors using a search algorithm that includes multiple subsets of the weighted predictor variables and their interactions. Typically, a large number of such competing variable subsets are compared with one another based on some measure of model fit. In addition, so as to reduce the likelihood of finding locally optimal results that will not generalize beyond the training sample, random changes to the variable subsets, not based on model fit, are also made. Most frequently the measure of model fit used to decide on the final weights for the main effects and interactions is a form of the familiar least squares criteria, i.e., the best fitting model is one that minimizes the difference between the observed and predicted outcome values. This method of ascertaining fit in NNET is known as back-propagation, where the difference between actual and predicted outputs is used to adjust the weight values. The quantity to be minimized in this approach is

(6) $$\sum_{i\;=\;1}^{N}{\left(y_{i}-{\hat{y}}_{i}\right)}^{2}$$

where *y*_*i*_is the observed value of the outcome variable for individual *i* and $\hat{y}$_*i*_ is the model predicted value for individual *i*. The weights and interactions of the variables are selected so as to minimize this value.

There are a number of NNET models available for use, with perhaps the most common of these (and the one utilized in this study) being the feed-forward back propagation network with one hidden layer (Garson, 1998). This particular NNET architecture uses the least squares minimization method described above in order to obtain weights for the inputs, which are the predictor variables. This model includes what is known as a hidden layer, which is analogous to one or more interactions in the more familiar regression context (Garson, 1998). It should be noted, however, that nodes in this hidden layer can be much more complicated than the interactions one might see in a standard linear model, involving complex combinations of the weighted predictor variables (Schumacher et al., 1996). Finally, the inputs and hidden layers are used in conjunction with the weights in order to obtain the predicted outputs, which, in this case is group membership, leading to the use of the logistic form of the model.

A potential strength of NNET models is that they can identify complex interactions among the predictor variables in the hidden layer that other methods will ignore (Marshall and English, 2000). Indeed, NNETs not only search for optimal weights for the main effects much as LR might, but they also examine various combinations of the predictors beyond the simple interactions typical in the regression context, which most of the other methods included in this study do not do. Therefore, whereas in regression it is common to express the interaction of two predictors simply as their product, or to square or cube a single predictor variable if its relationship with the response is believed to be non-linear, a NNET will create hidden nodes as weighted products of potentially several variables, some of which are also raised squared or cubed, for example. This construction allows the hidden nodes to be influenced by the predictors in varying degrees. If, for example, two variables interact and none of the others play a role, then the hidden layer would be represented by large weights for each of the two and near 0 weights for the others. On the other hand, a hidden layer could be thought of as the combination of several of the predictors with some contributing slightly more and thus having slightly larger weight values.

As with several of the techniques described above, NNETs have a tendency to overfit the training data (Schumacher et al., 1996). In order to combat this problem, most NNET models apply weight decay, which penalizes the largest weights found by the original NNET analysis, in effect assuming that very large weights are at least partially driven by random variation unique to the training data. An alternative, known as weight elimination, reduces the smallest weights to values very near 0, essentially pruning away small weights under the assumption that they represent random variation only.

## Methods

### Misclassification conditions

**Figure 1:**
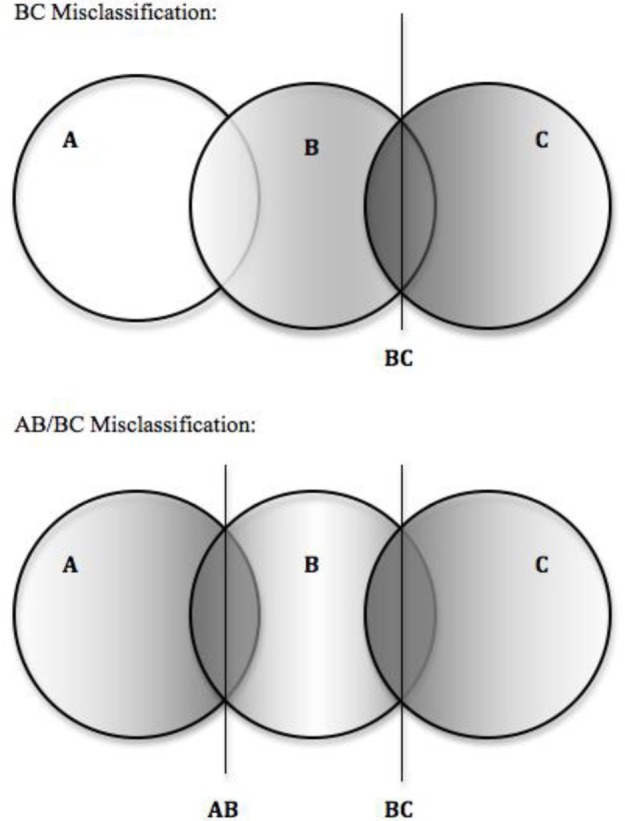
**Depiction of simulated three group misclassification situations**. Note. Groups A, B, and C fall along a continuum such that *X*_*A*_ < *X*_*B*_ < *X*_*C*_. Darker shaded regions represent higher probability of misclassification.

In order to study the impact of training group misclassification on classification accuracy in the three group case, two different situations of three group misclassification were simulated (see Figure 1). Situation one, we will term BC misclassification. BC misclassification simulates the situation where misclassification happens only between two of the three groups. For example, in diagnosis of autism spectrum disorders, three groups can be conceptualized: children without an autism spectrum disorder, children with Asperger's Syndrome, and children with an autism spectrum disorder. It might be rare to misclassify children without an autism spectrum disorder as having Asperger's or autism, however, misdiagnosis between Asperger's and autism would be possible. Thus, this might be a situation where BC misclassification might occur.

Situation two we will term AB/BC misclassification. AB/BC simulates the situation where misclassification of all three groups does not occur but misclassification of adjacent groups is likely. This type of misclassification can be related to classification or diagnosis when classifications occur along a continuum. For example, refer to the previous discussion of cut-score misclassification in educational settings. Often in educational situations, a test may use cut-points to classify a student as “failing,” “proficient,” or “exceeds standards.” Each of these classifications is based upon a continuous test score, thus it would be unlikely to mistake a “failing” student as “exceeds standards.” When students score near the cut-points, however, it may be difficult to differentiate between “failing” and “proficient” or between “proficient and “exceeds standards.” Thus this might be a situation where AB/ BC misclassification might occur.

### Data generation

Generation and analysis of misclassified data was accomplished using the R statistical software program (R Development Core Team, 2007). Data were generated to meet the specific criteria listed in Table 1, with non-differential misclassification. In order to generate the data, true group membership was first assigned to each simulated subject. Then, cases nearer to the predetermined cut-points on the predictor variable were simulated as more likely to be misclassified than cases lying further away (Lathrop, 1986; Dwyer, 1996). In other words, cases with a relatively low probability of belonging to their initially assigned group were more likely to be misclassified than were those with a higher probability of their initial group membership. To achieve this type of misclassification, a random number between 0 and 1 was generated for each case and compared to a scaled cumulative probability of the case based on its score for the predictor variable. If the cumulative probability of group membership was lower than the randomly generated value, the case was then misclassified as belonging to the group for which it had the next highest probability of membership. Thus, cases with lower probabilities of initial group membership had a greater likelihood of being misclassified. When this procedure is used with no scalar adjustment, approximately 50% of the cases will be misclassified every time. However, by multiplying the location in the probability distribution by an appropriate scalar, *k*, the percent of data misclassified can be controlled so that a specific proportion of cases are misclassified. The appropriate scalar values corresponding to the 0%, 10%, 20%, and 30% were found through a mathematical proof (see Appendix of Holden and Kelley, 2010). Once the data were simulated to include misclassification, they were analyzed with each of the seven statistical classification analyses and results saved in terms of overall percent correct classification, and percent of each group correctly classified. A total of 1000 replications were simulated for each combination of simulation conditions in Table 1, which were completely crossed with one another. Simulation code is available from the authors upon request.

**Table 1:** **Simulation conditions for the single predictor three group case**.

**Data conditions**	
Type of 3 group overlap	BC, AB/BC
Population variance	1
Manipulated variables	
Statistical analysis method	LDA, QDA, LR, CART, GAM, NNET, MIXDA, RF
Percent misclassified (%)	0, 10, 20, 30
Sample size	150, 1500
Sample size ratio	50:50:50, 25:25:100, 25:100:25, 100:25:25
Standardized mean diff.	0.2, 0.5, 0.8, 1.6

## Results

### Overall misclassification

In order to determine which of the manipulated factors were significantly related to the overall misclassification rates, a full factorial repeated measures analysis of variance (ANOVA) was used. For each replication in the simulation, the outcome was overall misclassification, the repeated measures factor was method of classification, and the between subjects factors were sample size, group size ratio, percent of subjects misclassified, and level of group separation. In addition to the hypothesis test, the η^2^ effect size was also used. This statistic expresses the proportion of variation in the outcome that is accounted for by each term in the ANOVA model. In order for a main effect or interaction to be considered important in the context of this study, it must be statistically significant and must have η^2^of 0.1 or greater. It should also be noted that the logistic regression results were so similar to that of the linear discriminant analysis that they will not be shown and only the linear discriminant analysis results will be presented.

**Figure 2:**
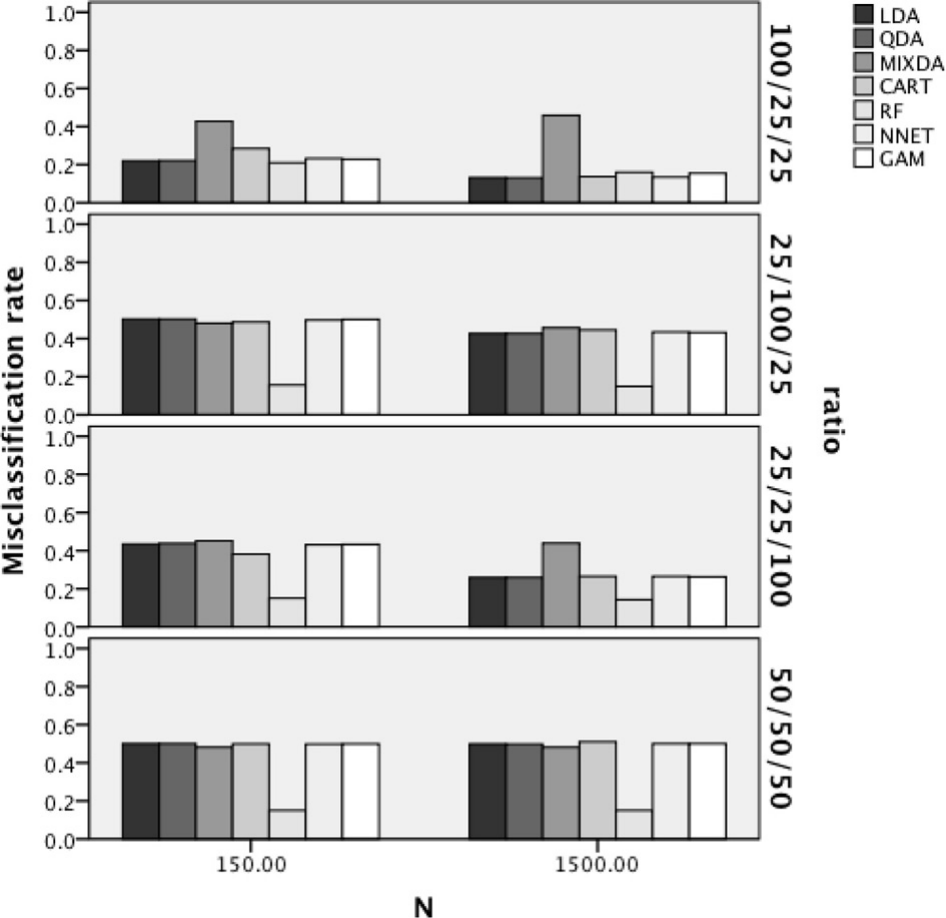
**Overall misclassification rates by method, sample size, and group size ratio**.

The results of the ANOVA for the overall misclassification rates indicated that the interaction of method X sample size X group size ratio [*F*_(18,312)_ = 14.415, *p* < 0.001, η^2^ = 0.454], the interaction of method X misclassification proportion, [*F*_(18,312)_ = 20.814, *p* < 0.001, η^2^ = 0.546] and the interaction of method X group separation [*F*_(18,312)_ = 9.563, *p* < 0.001, η^2^ = 0.353], were all statistically significant with η^2^greater than 0.1. Figure 2 includes the overall misclassification rates by method, sample size, and group size ratio. For ease of interpretation, when group size ratio is described it will always be listed as *n*_*A*_/*n*_*B*_/*n*_*C*_. Across both sample size and group size ratio, RF had the lowest misclassification rates across methods. In addition, for the 100/25/25 ratio condition all of the methods had comparable error rates, with the exception of MIXDA, which had a higher misclassification rate than the other methods. For both the 100/25/25 and 25/25/100 sample size conditions, the misclassification rates were lower for all methods except MIXDA and RF in the larger sample size case, while for the 25/100/25 and 50/50/50 conditions misclassification rates for all methods were comparable across sample sizes.

**Figure 3:**
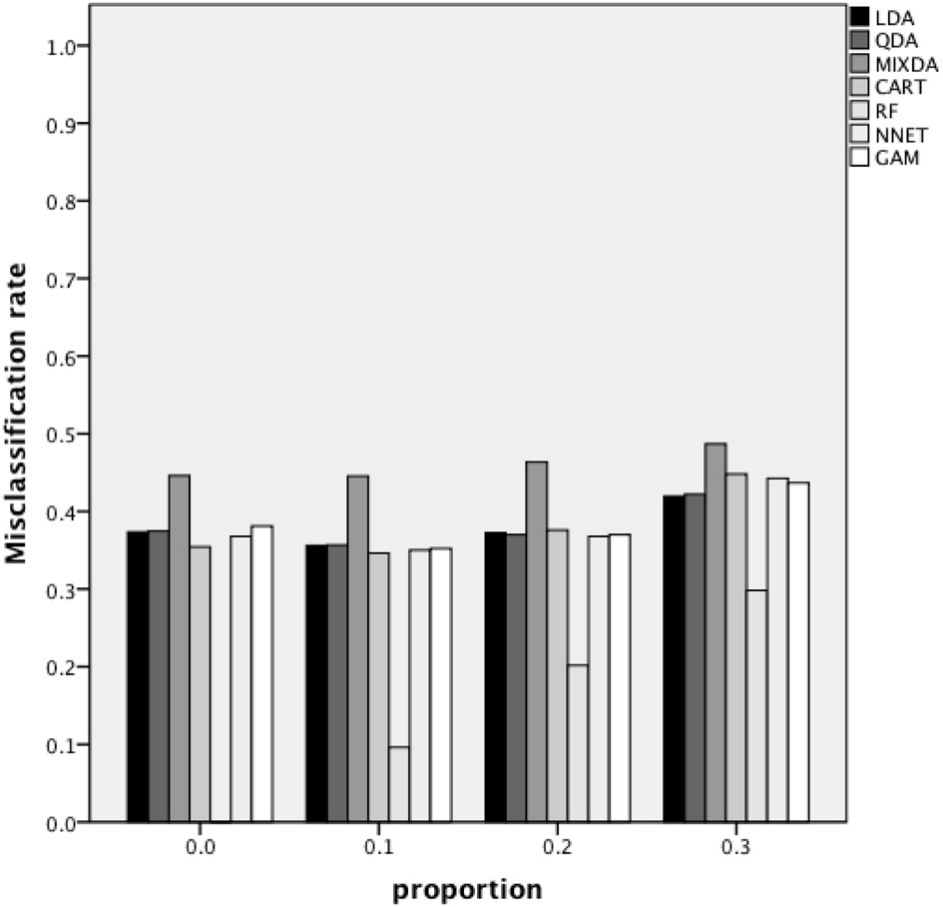
**Overall misclassification rate by method and proportion of cases initially misclassified**.

**Figure 4:**
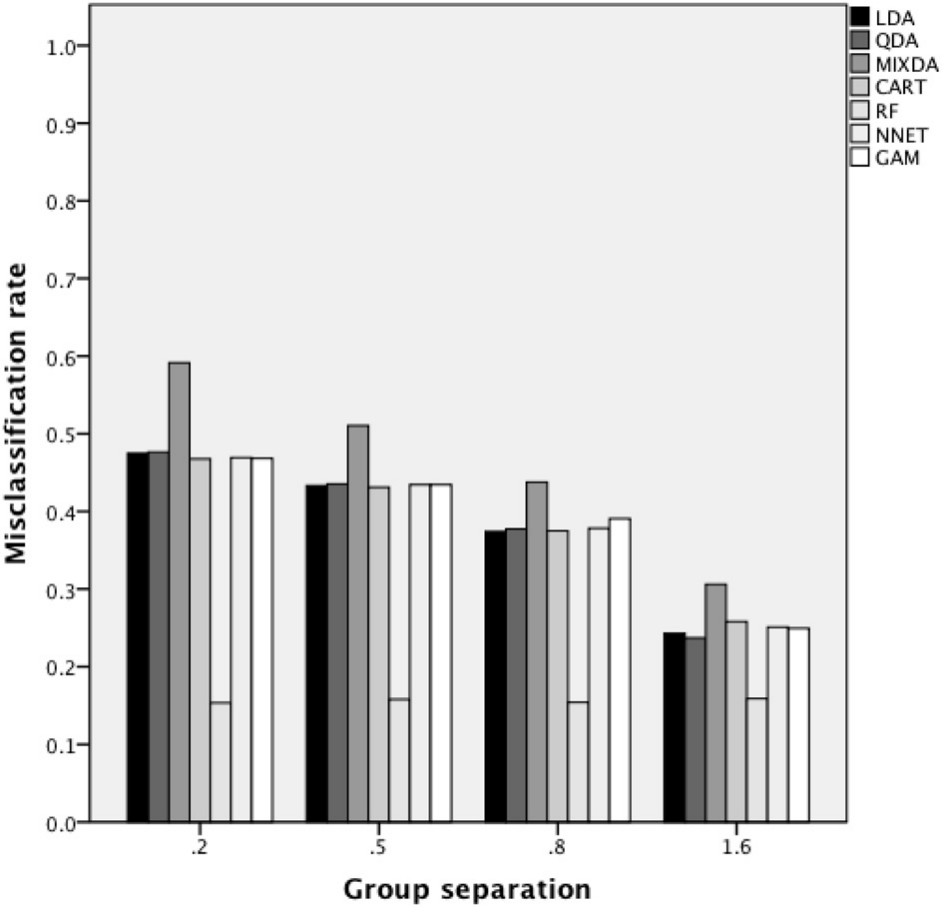
**Overall misclassification rate by method and group separation**.

Figure 3 includes the misclassification rates for method by misclassification proportion. Again, across proportion of cases misclassified, the misclassification rate for RF was the lowest across methods, while that of MIXDA was the highest. The other methods studied here all presented similar rates of misclassification across conditions. In addition, for RF there was an increase in the misclassification rate concomitant with increases in the proportion of cases that were initially misclassified. In contrast, for the other methods studied here, the misclassification rate did not increase until the proportion of cases originally misclassified reached 0.3. In other words, there appears to be a threshold between 0.2 and 0.3 above which the proportion of cases originally misclassified has an impact on the misclassification rates of most of the methods studied here, but below which no such effect is seen. Figure 4 shows the misclassification rate of the methods by the level of group separation. For all of the methods an increase in the level of group separation resulted in a decrease in the misclassification rates, with the exception of RF, for which the misclassification rate was very consistent (and the lowest) across levels of group separation. Just as RF yielded the lowest rates regardless of group separation, MIXDA yielded the highest, with the other methods performing similarly.

### Group misclassification

In addition to investigating the overall rate of misclassification, we also examined misclassification of the three groups individually. As with the overall results, repeated measures ANOVA was used to identify main effects and interactions of the manipulated factors that were both significantly related to the misclassification rates and yielded effect size values of 0.1 or greater. In this case, the analyses were run separately for each of the three group specific misclassification rates. The results of these three group specific analyses were qualitatively the same; i.e., the same model terms were identified as important by the criteria outlined above in terms of their impact on the misclassification rates of each group. Therefore, ANOVA results for group 1 only are presented here. The interactions of method by each of group size ratio

[*F*_(18, 210)_ = 8.603, *p* < 0.001, η^2^ = 0.424], misclassification proportion

[*F*_(18, 210)_ = 11.286, *p* < 0.001, η^2^ = 0.492], and group separation

[*F*_(24, 284)_ = 7.86, *p* < 0.001, η^2^ = 0.399] were found to be both statistically significant and to have η^2^ ≥ 0.10. In addition, the main effect for sample size was also statistically significant with η^2^ ≥ 0.10, [*F*_(1, 73)_ = 30.018, *p* < 0.001, η^2^ = 0.291].

Table 2 includes these rates for each method by misclassification proportion, group size ratio, and group separation, respectively. Misclassification rates for all groups increased for nearly all of the methods as the degree of original misclassification increased. The exception to this pattern was MIXDA, for which misclassification rates increased in group 1 concomitantly with the proportion of originally misclassified cases, but saw very little if any increase in the misclassification rates for groups 2 and 3 under these same conditions. In addition, the groups differed with respect to the magnitude of increase in the misclassification rates. For example, LDA, QDA, CART, and RF all had increases of between 0.3 and 0.4 in misclassification rates for groups 1 and 2 across levels of misclassification proportion, whereas NNET and GAM had increases for these groups in the range of 0.1–0.2. And, as mentioned above, MIXDA saw very little increase in the misclassification rates for these groups across values of misclassification proportion. For most of the methods misclassification rates were lowest for group 1, with the exception of MIXDA, which had comparable rates for groups 1 and 3, and RF which displayed comparable misclassification rates for all three groups.

**Table 2:** **Group misclassification percentage by method, misclassification proportion, group size ratio, and group separation**.

		**LDA**			**QDA**			**MIXDA**			**CART**			**RF**			**NNET**			**GAM**		
	**Group**	**1**	**2**	**3**	**1**	**2**	**3**	**1**	**2**	**3**	**1**	**2**	**3**	**1**	**2**	**3**	**1**	**2**	**3**	**1**	**2**	**3**
Misclass proportion	0	0.191	0.826	0.766	0.197	0.818	0.528	0.389	0.682	0.382	0.201	0.759	0.501	0.000	0.000	0.000	0.570	0.800	0.613	0.560	0.799	0.593
	0.1	0.205	0.695	0.876	0.208	0.707	0.616	0.388	0.649	0.362	0.241	0.644	0.579	0.096	0.098	0.098	0.513	0.730	0.629	0.409	0.678	0.629
	0.2	0.251	0.568	0.948	0.254	0.584	0.745	0.420	0.634	0.368	0.314	0.521	0.692	0.201	0.205	0.205	0.589	0.713	0.725	0.546	0.648	0.770
	0.3	0.334	0.425	0.979	0.346	0.424	0.835	0.461	0.649	0.372	0.427	0.384	0.790	0.299	0.298	0.298	0.760	0.751	0.821	0.719	0.701	0.888
Group size ratio	100/25/25	0.067	0.814	0.965	0.066	0.818	0.802	0.451	0.572	0.350	0.117	0.747	0.767	0.178	0.187	0.187	0.699	0.890	0.822	0.643	0.860	0.865
	25/100/25	0.312	0.546	0.875	0.319	0.553	0.648	0.401	0.684	0.380	0.359	0.520	0.625	0.153	0.153	0.153	0.585	0.694	0.668	0.520	0.649	0.677
	25/25/100	0.132	0.734	0.917	0.138	0.738	0.717	0.421	0.632	0.384	0.175	0.642	0.626	0.147	0.146	0.146	0.596	0.818	0.745	0.553	0.793	0.776
	50/50/50	0.442	0.426	0.850	0.453	0.431	0.612	0.399	0.704	0.363	0.511	0.404	0.597	0.150	0.150	0.150	0.583	0.616	0.599	0.544	0.544	0.620
Group separation	0.2	0.337	0.677	0.945	0.345	0.669	0.932	0.573	0.673	0.630	0.390	0.586	0.889	0.154	0.152	0.152	0.858	0.886	0.934	0.872	0.886	0.930
	0.5	0.277	0.681	0.907	0.288	0.677	0.833	0.439	0.776	0.409	0.314	0.620	0.784	0.158	0.159	0.159	0.735	0.840	0.862	0.698	0.796	0.862
	0.8	0.230	0.655	0.869	0.239	0.657	0.685	0.367	0.708	0.296	0.270	0.609	0.649	0.154	0.158	0.158	0.597	0.764	0.735	0.477	0.701	0.730
	1.6	0.149	0.463	0.872	0.145	0.494	0.305	0.286	0.451	0.150	0.230	0.457	0.267	0.158	0.160	0.160	0.254	0.496	0.275	0.198	0.429	0.385

The group misclassification rates for each method by the group size ratio appear in Table 2. These results paint a divergent picture among the methods in terms of their relative accuracy. For example, when the groups were of equal size, LDA and QDA both displayed the lowest misclassification rates for group 1. On the other hand, in this same condition, CART had the lowest rate for group 2, while GAM displayed lower rates for groups 1 and 2 both than for group 3, and MIXDA had lower rates for groups 1 and 3 than for group 2. Error rates for the three groups were comparable to one another for both RF and NNET in the equal group ratio condition. A similar theme of divergent results was in evidence for the other group size ratio conditions, so that no general pattern emerged across the methods. In general, RF yielded the lowest rates across conditions, however, regardless of the ratio. In addition, for several of the methods, the larger group tended to be favored in terms of misclassification rates, though this pattern was not universal.

Finally, the misclassification rates by degree of group separation also appear in Table 2. For all methods, the misclassification rates declined as the level of group separation increased in value, with the exception of for RF. In this latter case, misclassification rates were very consistent across levels of group separation. In addition, with the exception of GAM, the largest decrease in misclassification rates occurred for group 3 when group separation increased. Group 3 had the highest misclassification rates when group means had lower levels of separation. In the case of GAM, the greatest decline in misclassification rates with increasing group separation occurred for group 1. For all methods with the exception of RF, the smallest decrease in misclassification rates occurred for group 2. Finally, across levels of group separation, RF had the lowest group specific misclassification rates, with a few exceptions at the highest degree of separation, in which case its rates were comparable to those one or the other of the methods for one or the other of the groups (e.g., LDA and QDA for group 1). With respect to sample size, for all methods and all groups, misclassification rates were lower in the larger sample size condition.

## Discussion

In summary, results of the simulation studies presented here suggest random forests to be a very powerful method to consider when misclassification is likely to exist in training data. Interestingly, random forests appeared to be the most affected by data misclassification. Regardless of the amount of misclassification present in the data, however, it still provided the most accurate classifications. In contrast, random forests appeared only marginally affected by study characteristics such as sample size, group size ratio and effect size. Taken together, these results demonstrated that random forests yield the lowest misclassification rates of the methods studied here, even in cases when some individuals were initially misclassified. In most cases, CART provided the second most accurate classification accuracy in the face of data misclassification. The gap between random forests and CART, however, was generally quite large with random forests having a large advantage. In examining the more traditional forms of classification we find LDA and QDA to be less accurate than random forests and CART when classifying in the face of misclassified training data. However, they perform relatively similarly to GAM and Neural Networks. Mixture discriminant analysis generally produced the poorest classification accuracy across conditions.

This study also supports the findings of previous research indicating that the negative impact of initial misclassification is, to some degree, ameliorated by other sample factors. In particular large sample size, greater group separation, and the ratio of group sizes can have a profound impact on the classification accuracy of the techniques studied here. Increased sample size, effect size and group size ratio tend to increase overall classification accuracy. It should be noted, however, that when group size ratio increases, this can have a disparate impact on the group specific classification accuracy which is an important consideration.

### Conclusions

Classification of individuals into groups based on one or more variables is very common practice in social science research. For example, in this age of school and teacher accountability when many important decisions are based on whether students, teacher or schools are classified as “passing” or “failing” it is crucial that such classifications are as correct as possible. In addition, individuals are quite frequently classified as having a learning disability, or a psychological malady such as depression or anxiety based on their score(s) on one or more instruments. In the context of higher education, students are granted admittance to college in large part based upon their performance on entrance examinations such as the SAT or ACT. In all of these instances, classifications are frequently made using cut-points on a continuous variable (e.g., achievement test score, intelligence test, anxiety inventory, college entrance examination). However, it is well known that using such cut-point methods for this purpose is likely to result in an initial misclassification of group membership (Lathrop, 1986; Dwyer, 1996). Thus, if this initial grouping is to be used for creating a prediction algorithm for accurately classifying future individuals, such training group misclassification can be particularly problematic. In such cases it becomes particularly relevant to ascertain which classification algorithms might be most accurate and least influenced by initial misclassification.

### Directions for future research

This study aimed to fill some of the gaps in the literature, however, there is still a lot to learn. This study only looked at two potential ways three-group misclassification could occur. However, there are conceivably many different and complex ways misclassification could arise, thus in this respect we have only just brushed the surface. There are also many other variables which could impact classification accuracy in the presence of misclassification. Assumption violations, strength and number of predictors or misclassification of predictor variables are just a few possible alternative factors to consider. However, in the meantime, we hope that the results of this study provide educational researchers, practitioners and policy makers engaged in classification practices, research or decisions based on classifications with valuable information regarding the impact of misclassification on subsequent classification accuracy as well as provide sound advice for choice of statistical classification technique based on the situation at hand.

### Conflict of interest statement

The authors declare that the research was conducted in the absence of any commercial or financial relationships that could be construed as a potential conflict of interest.
